# Breast Cancer in the Personal Genomics Era

**DOI:** 10.2174/138920210791110951

**Published:** 2010-05

**Authors:** Rachel E. Ellsworth, David J. Decewicz, Craig D. Shriver, Darrell L. Ellsworth

**Affiliations:** 1 Clinical Breast Care Project, Henry M. Jackson Foundation for the Advancement of Military Medicine, Windber, PA, USA; 2 Clinical Breast Care Project, Walter Reed Army Medical Center, Washington, DC, USA; 3 Clinical Breast Care Project, Windber Research Institute, Windber, PA, USA

**Keywords:** Breast cancer, personal genomics, genetic tests, gene expression, risk assessment.

## Abstract

Breast cancer is a heterogeneous disease with a complex etiology that develops from different cellular lineages, progresses along multiple molecular pathways, and demonstrates wide variability in response to treatment. The “standard of care” approach to breast cancer treatment in which all patients receive similar interventions is rapidly being replaced by personalized medicine, based on molecular characteristics of individual patients. Both inherited and somatic genomic variation is providing useful information for customizing treatment regimens for breast cancer to maximize efficacy and minimize adverse side effects. In this article, we review (1) hereditary breast cancer and current use of inherited susceptibility genes in patient management; (2) the potential of newly-identified breast cancer-susceptibility variants for improving risk assessment; (3) advantages and disadvantages of direct-to-consumer testing; (4) molecular characterization of sporadic breast cancer through immunohistochemistry and gene expression profiling and opportunities for personalized prognostics; and (5) pharmacogenomic influences on the effectiveness of current breast cancer treatments. Molecular genomics has the potential to revolutionize clinical practice and improve the lives of women with breast cancer.

## INTRODUCTION

Breast cancer is the most frequently occurring cancer and the leading cause of death in women between 20 and 59 years of age in the United States [1]. Over the last fifty years, the incidence of breast cancer has increased dramatically, such that today one in eight women are expected to develop breast cancer during her lifetime. Last year in the United States, more than 40,000 women died from breast cancer [2] and the cost of treatment for breast cancer patients exceeded $8 billion [3].

Breast cancer has a complex etiology where susceptibility is influenced by both environmental and genetic factors. Considerable experimental and epidemiological evidence suggests that lifetime exposure to endogenous hormones, notably estrogens and androgens, promotes breast carcinogenesis. In population-based studies, factors related to increased estrogen exposure throughout a woman’s lifetime, such as early menarche, late menopause, use of oral contraceptives, and hormone replacement therapy, have been associated with a ~2-fold increase in breast cancer risk among premenopausal women [4-6]. Other risk factors including age, family history, late age (>30 years) at first pregnancy or never being pregnant, and high breast density [7], as well as modifiable risk factors such as nutrition [8], exercise, and alcohol/tobacco use are also important in defining risk for breast cancer.

Heterogeneity in clinical, pathological, and molecular characteristics makes breast cancer a challenging disease to manage. Pathological characterization of breast carcinomas includes a number of variables such as tissue architecture, cellular differentiation, and size/presence of local or distant metastasis, which influence disease progression, risk assessment, and prognosis. Other variables including estrogen receptor (ER), progesterone receptor (PR), and epidermal growth factor receptor-2 (HER2) expression vary widely among breast cancer patients and thus are routinely assessed as a component of standard care for determining the most effective treatment. At the molecular level, breast cancer has been found to exhibit extensive variability, with at least five tumor subtypes identified by patterns of gene expression [9, 10]. In women with similar pathological characteristics, molecular heterogeneity of breast disease may cause clinical outcomes to vary widely even though patients receive identical treatments [11].

The extensive molecular and pathological diversity observed in breast cancer patients suggests that breast cancer is not a homogeneous disease that can be effectively managed by a “standard of care” approach. Breast cancer may be more appropriately defined as a myriad of diseases characterized by variability in developmental pathways, propensity to metastasize, and response to treatment that can only be successfully treated by regimens targeted to individual patients. Personalized medicine provides care and treatment based on fixed and modifiable risk factors unique to each patient, as well as pathological and molecular characteristics that make each breast carcinoma unique. In this review, we critically examine the role of inherited and somatic genomic variation in breast cancer, outlining the predictive utility of susceptibility variants, extent and potential information content of cancer genomics, and the promise of personalized medicine and personalized oncology.

## HEREDITARY BREAST CANCER

### Identification of Susceptibility Genes

The idea that breast cancer has a familial or inherited component was proposed as early as 1757 when Le Dran described a 19-year old woman with breast cancer whose grandmother and maternal uncle died of breast cancer [12]. In 1866, Broca characterized a family with ten women across four generations who were affected with breast cancer, and provided sufficient data to create a family pedigree that clearly showed the heritable nature of the disease [13]. More recently, twin studies and segregation/risk analysis have provided additional evidence that the development of breast cancer has a genetic component [14-17].

Identification of genes associated with the development of breast cancer is complicated by the co-occurrence of sporadic and heritable breast cancer within families. Because breast cancer affects one in eight women, approximately 11% of families will contain more than one female with breast cancer [18], but it may be challenging to distinguish disease attributable to an inherited cancer susceptibility gene from chance clustering of sporadic breast cancer cases within a family. To identify breast cancer susceptibility genes, large extended families meeting stringent criteria for defining heritable breast cancer were needed. Using this approach, 23 extended Caucasian families containing 146 individuals with breast cancer including early onset cases, bilateral disease, and/or male breast cancer were assembled. Forty percent of these families showed strong linkage to a marker on chromosome 17q21 [19]. In 1994, a novel gene with a zinc-finger domain and wide tissue expression, including breast and ovary, was identified as the breast cancer 1 gene (BRCA1) [20].

Despite the strong contribution of BRCA1 to hereditary breast and ovarian cancer, mutations in BRCA1 do not account for all cases of inherited breast cancer, implicating the existence of a second major susceptibility gene. Using techniques similar to those used in the discovery of BRCA1, a novel gene on chromosome 13q12-q13 was identified as BRCA2 in 1995 [21, 22].

Following the identification of BRCA1 and BRCA2, screening tests were developed to identify mutation carriers and families at risk for hereditary breast and ovarian cancer. Screening is now recommended for women with breast cancer diagnosed at an early age, women with bilateral breast cancer, women with a family history of breast and ovarian cancer involving multiple family members (including males), and women of Ashkenazi Jewish ancestry [23]. Currently in the United States, Myriad Genetics (www.myriad.com) is the sole provider of BRAC*Analysis*^®^, a direct sequencing approach to detect mutations in BRCA1 and BRCA2.

### Patient Management

Clinical management of BRCA mutation carriers may include surgical intervention, chemoprevention, or increased surveillance. Although prophylactic mastectomy is the most effective intervention for BRCA1 and BRCA2 carriers, reducing the risk of developing breast cancer by 90% [24], mastectomy is a highly invasive procedure with adverse physical and potentially devastating psychological effects. Thus, other non-surgical methods have been developed for detection and risk reduction [25]. As tamoxifen in BRCA mutation carriers with breast cancer has been shown to significantly decrease the rate of contralateral disease [26], chemoprevention may be considered as part of an overall risk management program. In addition, magnetic resonance imaging (MRI) is more sensitive than mammography for detecting malignancy in BRCA carriers [27]. The International Consensus Conference on Breast Cancer Risk, Genetics, and Risk Management recently advocated use of both mammography and MRI at alternating six-month intervals for screening BRCA mutation carriers [28].

BRCA status appears to confer a unique tumor phenotype that may contribute to poorer outcomes in patients with breast cancer. Primary breast tumors from BRCA1 mutation carriers have unique pathology, including high histological grade, atypical medullary histotype, high rates of cellular proliferation, pushing margins and infiltrating lymphocytes, and are frequently ER, PR, and HER2 negative (triple negative) [29]. These clinical attributes contribute to the “basal-like” phenotype of BRCA1 breast carcinomas and may influence tumor behavior and aggressiveness. Some studies have observed lower survival in BRCA1 mutation carriers compared to non-carriers [30, 31], suggesting poorer outcomes in patients with BRCA1 mutations; however, other studies have found no evidence of significant differences in long-term survival [32, 33].

The availability of genetic testing for BRCA mutations is potentially valuable in surgical decision making for newly diagnosed breast cancer patients, as approximately one-half of patients who are discovered to carry BRCA1/2 mutations are likely to choose bilateral mastectomy compared to only one-fourth of non-carriers [34]. At present, BRCA mutation status is not considered when making recommendations for systemic therapy because response to such therapies has not been shown to differ between carriers and non-carriers [28]. However, alterations in DNA repair caused by defects in BRCA1 and BRCA2 may enhance the sensitivity of BRCA-positive tumors to platinum agents such as cisplatin and carboplatin, which cross-link DNA and interfere with replication [35]. Similarly, BRCA dysfunction sensitizes cells to poly(ADP-ribose) polymerase (PARP) inhibitors, leading to chromosomal instability, cell cycle arrest, and apoptosis in BRCA1 and BRCA2 deficient cells [36]. Clinical trials are currently underway to determine the efficacy of PARP inhibitors in treating BRCA deficient breast tumors, which may open new avenues for less toxic therapies that target particular DNA repair pathways in BRCA1 and BRCA2 mutation carriers [37].

#### Genomic Variability Among BRCA Carriers

Identification of BRCA1 and BRCA2 has improved clinical management of some individuals with hereditary breast and ovarian cancer, but personalized care is not yet available for mutation carriers. Important barriers to effective treatment include allelic heterogeneity, environmental effects, and genetic modifiers. Allelic heterogeneity, where different mutations in the BRCA1 and BRCA2 genes show variability in penetrance, has been associated with different risks for developing disease [38, 39]. Although many women with BRCA mutations have a high probability of developing breast cancer, 15% of BRCA1-positive women and 20% of BRCA2-positive women will never develop breast cancer [40]. Likewise, environmental factors such as exercise and body weight, environmental exposures [41], contraceptive and hormone use, and reproductive history may influence tumor development in BRCA carriers [42]. Modifier genes are believed to contribute to variability in cancer risk, but the identification of genetic modifiers has been difficult. To date, only a single variant (-135G>C) in the recombination protein A (RECA or RAD51) gene has been confirmed as a genetic modifier in BRCA2 carriers [43]. The androgen receptor (AR), amplified in breast cancer 1 (AIB1), and aurora kinase (AURKA) genes have been implicated as genetic modifiers in BRCA carriers, but remain only candidates as positive results have yet to be replicated [44].

To hasten the identification of genetic modifier genes, the Consortium of Investigators of Modifiers of BRCA1 and BRCA2 (CIMBA) was established in 2005 as a “consortium of consortia”. With the collection of clinical data and genetic material from more than 15,000 BRCA1 and BRCA2 patients from around the world, genetic studies from CIMBA should have sufficient power to identify modifier genes [45]. Identification of genetic modifiers that influence risk of developing breast and/or ovarian cancer is needed to refine individual risk estimates and guide treatment options, including the need for prophylactic mastectomy and/or oophorectomy, in a personalized fashion.

Mutations in the BRCA1 and BRCA2 genes account for the majority of families with six or more cases of early-onset breast and/or ovarian cancer; however, many families with a high incidence of breast cancer have no detectable mutations in BRCA1 or BRCA2 [46], suggesting the existence of additional breast cancer susceptibility genes. Although genes associated with increased susceptibility have been identified, the majority of causative breast cancer genes remain unknown [47] — see Fig. (**1**). Confounding factors such as (1) heterogeneous phenotypes attributable to several unidentified BRCA genes, each accounting for a small number of cancer cases; (2) genes influencing development of other types of cancer in addition to breast that may not be linked to breast cancer; and (3) weakly penetrant genes that are heritable but resemble sporadic breast cancer in appearance [48] make the identification of high-risk breast cancer genes difficult, but critical to providing personalized and effective care to patients with hereditary and/or familial breast cancer.

## RISK ASSESSMENT FOR SPORADIC BREAST CANCER

### Risk Estimation

Inherited mutations have been associated with 10-15% of all breast cancer cases; however, disease etiology in the majority of women appears to be sporadic, lacking a significant family history. Because sporadic breast cancer may be influenced by a number of lifestyle and environmental factors as well as common low-risk variants in a number of genes, several models have been developed in an attempt to quantify individualized breast cancer risk:

- The Gail model measures risk based on patient age, age at menarche, number of prior breast biopsies, age at first live birth, and number of first degree relatives affected by breast cancer [49].
- The Claus model estimates risk based on the number of affected relatives and their respective age at diagnosis [50].
- The BRCAPRO model calculates risk of developing breast cancer based on the probability of carrying a BRCA1 or BRCA2 mutation [51].

These models have been widely used to predict risk and direct patient care, but each model has limitations because no model accounts for the spectrum of risk factors influencing breast cancer. For example, the Gail model considers only first-degree relatives without regard to age at diagnosis or presence of ovarian cancer, thus potentially underestimating genetic risk. The Claus and BRCAPRO models only consider family history, potentially underestimating risk in women with other risk factors [52]. In addition, these models were developed 10-20 years ago when incidence of breast cancer in the general population was lower than it is today — use of lower baseline risk estimates may contribute to an underestimation of current risk [53]. More recent models, such as the Tyrer-Cuzick model, utilize family history, endogenous estrogen exposure, and presence of benign disease to model breast cancer risk [54], but contributions from other factors such as mammographic breast density, weight gain, steroid hormone levels, and susceptibility genes have not been incorporated [55].

The discovery of the BRCA1 and BRCA2 genes advanced risk assessment in families affected by hereditary breast and ovarian cancer, but identification of molecular markers associated with increased breast cancer risk in patients without a family history of breast cancer has remained far more challenging. Without a strong family history, linkage approaches involving large pedigrees such as those used to identify BRCA1 and BRCA2 are not applicable. Sporadic breast cancer is not usually associated with other cancers such as ovarian or male breast cancer and unlike BRCA1-positive carcinomas, which exhibit specific histological characteristics, sporadic breast cancer cases comprise a vast array of phenotypes. Early approaches to identify sporadic breast cancer susceptibility genes compared the frequency of DNA variants in genes from molecular pathways believed to be involved in breast cancer development between cases with disease and healthy matched controls. An association study using candidate genes recently identified caspase-8 (CASP8) as a low-risk susceptibility gene where the major (H) allele of the D302H polymorphism had a protective effect on the development of breast cancer [56]. Despite success in identifying CASP8, candidate gene approaches have not been widely successful in identifying additional breast cancer susceptibility genes [57].

### Whole-Genome Approaches

Candidate gene approaches are rapidly giving way to genome-wide association studies (GWAS), which evaluate a dense array of genetic markers representing common variation throughout the genome. Completion of the human genome sequence and subsequent identification of single nucleotide polymorphisms (SNPs) now permits millions of informative SNPs across the genome to be assayed simultaneously. GWAS are useful for mapping genes of interest to small, localized regions of the genome and for detecting the effects of common (>5% minor allele frequency) alleles on disease risk [58]. Moreover, GWAS are performed without *a priori* knowledge of the underlying genetic defect(s), which may be advantageous since many genes identified through whole genome approaches were not previously suspected to influence the disease under investigation [59].

Recent GWAS have identified a number of loci that appear to be associated with breast cancer susceptibility (Table **1**). For example, the fibroblast growth factor receptor 2 (FGFR2), mitogen-activated kinase kinase kinase 1 (MAP3K1), lymphocyte-specific protein (LSP1), and trinucleotide repeat-containing 9 (TNRC9/LOC643714) genes, along with a 110 kb region of chromosome 8q24 have been associated with breast cancer in large studies involving thousands of subjects [60, 61]. Associations with other chromosomal regions — 2q35, 5p12, 6q22, and 16q12 — also have been reported [62-64]. Further analysis has shown that allelic variation at FGFR2, TNRC9, 8q24, 2q35, and 5p12 is associated with physiological characteristics of breast tumors, such as ER status [62, 64, 65], and specific FGFR2, MAP3K1, and TNRC9 variants may interact with BRCA1 and BRCA2 mutations to increase breast cancer risk [66].

Despite recent success in identifying genetic determinants of breast cancer, susceptibility alleles identified through GWAS are believed to account for only ~5% of breast cancer risk [67]. If future studies are to be successful in identifying additional low-risk susceptibility alleles and low-frequency, highly-penetrant variants [68], interactions between genes and environmental exposures must be assessed [69] and methods must be developed to evaluate mechanisms by which DNA variants in intronic or intergenic regions contribute to disease. As risk associated with susceptibility alleles may vary between racial/ethnic populations due to differences in frequency, patterns of disequilibrium, and interactions with environmental factors [60, 62, 70], sufficiently powered genetic studies in women from various ethnic groups are needed to improve risk reduction strategies for all women.

### Direct-to-Consumer Testing

New susceptibility variants identified by GWAS have not yet been incorporated into genetic tests with beneficial clinical utility for breast cancer patients. However, genetic analysis and risk assessment are available commercially through direct-to-consumer (DTC) testing. A number of for-profit companies offer personal genetic information based on DTC tests — the largest and most recognized companies include 23andMe (www.23andme.com), deCODEme (www.decodeme.com), Navigenics^®^ (www.navigenics.com), and Knome^®^, Inc. (www.knome.com/home) (Table **2**). For a fee of $99 to $99,500 consumers provide a blood, buccal, or saliva sample for targeted SNP analysis or whole-genome sequencing. Genetic information provided to the consumer varies greatly among companies, from trivial facts such as ear wax type to ancestry information to information on risk for disease [71, 72]. Although DTC tests epitomize “personalized genomics” by providing consumers with individual genotypes, critics note that the clinical utility of such tests is limited and often incongruent with marketing claims. Because information on family history and environmental exposures is usually not accounted for, DTC risk estimates may not be sufficiently accurate to enable consumers to make appropriate medical decisions [73, 74].

The majority of genetic risk assessments developed thus far focus on DNA variants; however, a new RNA-based signature has been developed for non-invasive breast cancer screening using peripheral blood samples. Although based on a small number of cases (n=24) and controls (n=32), a subset of 37 genes in the assay correctly classified 82% of patients [75]. Despite a relatively high misclassification rate, DiaGenic (www.diagenic.no) has since developed this gene expression signature into a clinical screening tool, currently available only in India as BCtect^™^ India.

## MOLECULAR CHARACTERIZATION OF BREAST TUMORS – PERSONALIZED PROGNOSTICS

### Pathological Characterization

Human breast carcinomas exhibit diverse pathological characteristics that are associated with different clinical outcomes, and thus are routinely used to guide treatment options. Accordingly, an accurate definition of prognosis is dependent on the ability to detect and quantify differences in tumor attributes, such as rates of proliferation and propensity to metastasize. Routine tumor evaluation currently includes: (1) histopathological classification; (2) grade determination; and (3) quantification of tumor size, surgical margin status, and lymph node involvement. Histopathological characterization, based on microscopic cellular morphology, classifies breast carcinomas into common subtypes (ductal or lobular carcinoma), which tend to have similar prognoses [76], or less common forms such as mucinous, tubular, and papillary (favorable prognosis) [77] or inflammatory breast cancer (poor prognosis) [78]. Increasing tumor size has long been associated with poor prognosis [79], but improved mammographic detection of smaller tumors has decreased the prognostic utility of tumor size [80]. Presence of positive surgical margins has been associated with local recurrence, but only 27% of patients with extensively positive margins will have recurrent disease [81, 82]. Likewise, the Nottingham Histological Score, widely used for assessing histological grade, is clinically useful for stratifying patients into low risk (low-grade disease, 95% five-year survival) and high risk (high-grade disease, 50% five-year survival) groups [83, 84], but the reliability of breast tumor grade in predicting survival is hampered by subjectivity associated with its assessment [85]. Axillary lymph node status is the most reliable predictor of survival, differentiating women who are likely to have >90% five-year survival (patients with negative nodes) from those who are likely to have <70% survival (women with nodal metastasis) [86]. Although these clinical attributes are currently the standard of care for breast cancer patients, many are imprecise in their ability to accurately predict outcomes.

### Immunohistochemistry

Molecular markers have the potential to provide additional prognostic information to supplement traditional pathological assessments for disease management in breast cancer patients. As mentioned above, traditional immunohistochemistry (IHC) markers routinely used in the classification of breast cancer include ER, PR, and HER2. Tumors positive for ER and PR expression frequently have low cellular proliferation rates, tend to exhibit lower histological grade, and are associated with more favorable prognosis [87]. ER and PR expression also is useful for identifying patients who will likely benefit from hormonal therapy, as women with ER and PR negative breast cancer do not gain a survival benefit from anti-estrogen tamoxifen [88].

The HER2 gene is a member of the epidermal growth factor receptor family with tyrosine kinase activity and is amplified at the DNA level and/or over-expressed in 15-25% of breast cancers. Carcinomas with amplified/over-expressed HER2 exhibit high histological grade and usually have a poor prognosis [89, 90]. Some patients with positive HER2 status (15-20%) are eligible to receive trastuzumab, a monoclonal antibody targeting HER2, in combination with standard chemotherapy [91].

Rigorous clinical studies have shown that evaluating ER, PR, and HER2 status provides additional prognostic information beyond that normally achieved by histological assessment alone. For example, breast carcinomas that are ER-negative, PR-negative, and do not have HER2 over expressed (triple negative) are marked by aggressive behavior, but because women with triple-negative disease are not eligible for tamoxifen or trastuzumab treatment, they usually have relatively low long-term survival [92]. Other markers such as nuclear antigen Ki67 are not routinely used to guide treatment selection, but hold great promise for monitoring the effectiveness of neoadjuvant chemotherapy and predicting recurrence-free survival [93-95].

Individual estimates of outcome using clinical and pathological characteristics of breast tumors, including age, menopausal status, co-morbid conditions, tumor size, number of positive lymph nodes, and ER status have been incorporated into a computer program, Adjuvant! Online (www.adjuvantonline.com/index.jsp), which is available over the Internet as a decision aid for patients and their physicians [96]. The program estimates the efficacy of endocrine therapy and chemotherapy as well as overall and disease-free survival in a user-friendly format that effectively brings patients into the decision-making process regarding personalized treatments.

Although IHC analysis of ER, PR, and HER2 is widely used in the pathological evaluation of breast tumors, additional molecular signatures involving multiple genes and/or proteins are desperately needed to more accurately classify tumors and guide treatment selection. Recently, a multi-gene IHC-based test known as MammoStrat^®^ (Applied Genomics, Huntsville, AL; www.applied-genomics.com/mammostrat. html) was developed to classify breast cancer patients into low-, moderate-, or high-risk categories for disease recurrence [97] (Table **3**). MammoStrat^®^ uses conventional paraffin-embedded tissue to assay five markers by IHC: tumor protein p53 (TP53) — known to play a central role in cell cycle regulation; *Hpa* II tiny fragments locus 9C (HTF9C) — involved in DNA replication and cell cycle control; carcinoembryonic antigen-related cell adhesion molecule 5 (CEACAM5) — aberrantly expressed in some cancers; nmyc downstream-regulated gene 1 (NDRG1) — may function as a signaling protein in growth arrest and cellular differentiation; and solute carrier family 7 (cationic amino acid transporter, y+ system), member 5 (SLC7A5) — mediates amino acid transport. The MammosStrat^®^ test may have utility for predicting patient outcomes, but currently requires five separate slides (one slide per antibody), which has the potential to show variability in staining intensity and scoring between patients.

### Gene Expression Signatures and Tumor Classification

With the sequencing of the human genome and identification of many human genes, whole-genome approaches using array-based methods have been developed to evaluate expression levels for thousands of genes in a single experiment. Initial studies of human breast carcinomas identified several distinct subtypes of breast cancer using large-scale gene expression profiling: (1) ER positive (luminal-like), characterized by high expression of many genes expressed in breast luminal cells; (2) basal-like; (3) HER2 positive; and (4) normal-like [9]. Further experiments subdivided the luminal (ER positive) tumors into luminal A and luminal B subtypes, and found that these five subtypes are useful in predicting relapse-free and overall survival, with the HER2 positive and basal-like subtypes having the shortest survival times [10].

To improve the clinical utility of molecular signatures for predicting outcomes in women with breast cancer, a new gene signature encompassing a larger number of genes has been identified. The Single Sample Predictor (SSP) signature is reported to identify the original five intrinsic subtypes, plus a new “IFN-regulated” subtype, characterized by high expression of interferon-regulated genes [98]. Likewise, the Breast Bioclassifier^™^ (University Genomics, Saint Louis, MO) is a commercial assay for classifying breast carcinomas by subtype. Expression levels for 55 genes provide subtype information and a continuous risk score to guide treatment options (www.bioclassifier.com/).

As gene expression signatures for classifying breast tumors likely reflect underlying biological characteristics of disease, a number of gene expression signatures focusing on specific molecular pathways have been developed. For example, tumorigenesis and wound healing share many similar physiological and molecular processes, including recruitment of inflammatory cells, stimulation of fibroblast and epithelial cell proliferation, cell migration, and angiogenesis. A “wound response” gene expression signature has been associated with metastatic progression and mortality in breast cancer patients [99], and may improve risk stratification independently of established clinicopathological risk factors [100]. If the wound response signature proves to be effective in identifying patients at high-risk of recurrence after breast conserving therapy, it may be useful as a diagnostic tool to identify patients who should be offered more aggressive treatments, such as increased radiation, or who should consider mastectomy rather than breast conserving therapy [101]. Likewise, breast carcinomas showing high levels of expression for hypoxia-related genes tend to exhibit p53 mutations, negative ER status, and high histological grade, and have been associated with lower overall and disease-free survival [102].

Gene expression profiles are increasingly being used as molecular tools to complement pathological evaluation and guide treatment options. To overcome inherent subjectivity in histological grading of breast tumors, the Gene expression Grade Index (GGI) was developed to summarize the similarity between gene expression profiles and tumor grade. The score attempts to classify low-grade and high-grade tumors, and to subdivide intermediate-grade tumors into low-grade, high-grade, or mixed-grade groups [103]. Further refinement of the gene expression grade index led to the Map*Quant* DX^™^ Genomic Grade assay (Ipsogen, Marseille, France; www.ipsogen.com/), marketed as the first microarray-based diagnostic test to measure tumor grade [104]. With the reported ability to classify ~80% of intermediate-grade breast tumors as either low-grade or high-grade, the Map*Quant* DX^™^ Genomic Grade assay may be useful in guiding treatment options, possibly sparing patients with low-grade (grade 1 or grade 1-like) tumors unnecessary treatments, while identifying those patients who would most likely benefit from chemotherapy.

### Gene Expression Signatures and Disease Risk

Molecular profiles are now being used more frequently as clinical tools to determine treatment for certain groups of patients by categorizing them into low-risk and high-risk groups. The MammaPrint^™^ assay (Agendia, Amsterdam, The Netherlands; www.agendia.com) is a 70-gene signature developed using tumor tissue from young women (<55 years of age) with node-negative disease, who either developed distant metastasis or remained disease-free after five-years [105]. Overall 10-year survival for the “poor-prognosis” signature is ~55%, while 10-year survival in women with the “good-prognosis” signature is 95%. The probability of being free from distant metastasis after 10 years is 51% for the poor prognosis and 85% for the good prognosis profile [106]. A second group of researchers subsequently developed a 76-gene profile (Rotterdam signature) that could identify breast cancer patients at high risk for distant recurrence. The signature could identify patients who developed distant metastases within five years when traditional prognostic factors were considered (hazard ratio 5.55, 95% CI 2.46–12.5) and could predict metastasis in both premenopausal and postmenopausal patients [107].

The gene expression signatures outlined above were refined from global expression profiling experiments involving thousands of genes and flash-frozen tumor specimens. An alternative approach relied on an extensive literature search to identify candidate genes (n=250) believed to be involved in disease development based on known function. Gene expression levels were assayed in 447 patients with ER-positive, node-negative breast cancer to identify a small subset of 16 genes (plus five reference genes) amenable to analysis by real-time-PCR (RT-PCR) on RNA isolated from formalin-fixed, paraffin-embedded (FFPE) specimens. The resulting 21-gene signature, known as Onco*type* DX^®^ (Genomic Health, Redwood, CA; www.genomichealth.com/) provides a probability of recurrence score for women with early stage (Stage I or II), ER-positive, node-negative breast cancer, and categorizes patients as low-, intermediate-, or high-risk. In validation studies using patients from the National Surgical Adjuvant Breast and Bowel Project (NSABP) clinical trial B-14 who received tamoxifen, the probability of distant recurrence at 10 years for the three risk categories was: low-risk — 6.8% (95% CI 4.0-9.6); intermediate-risk — 14.3% (95% CI 8.3-20.3); and high-risk — 30.5% (95% CI 23.6-37.4). Recurrence scores also correlated significantly with relapse-free interval and overall survival [108]. In a subsequent study, Onco*type* DX^™^ was used to assess the benefit of adjuvant chemotherapy in ER-positive, node-negative patients. Because the highest benefit was observed in patients with high-risk scores, while women with low-risk recurrence scores did not benefit from chemotherapy [109], Onco*type* DX^™^ may be useful in guiding treatment options in ER-positive, node-negative patients.

Clinical trials of the MammaPrint^™^ and Onco*type* DX^™^ assays are currently in progress. In the Microarray In Node-negative Disease may Avoid ChemoTherapy (MINDACT) trial, 6,000 node-negative women will be assigned to treatment groups based on risk stratification by traditional clinical-pathological factors (ADJUVANT! Online) and the MammaPrint^™^ molecular signature [110]. Patients classified as low risk by both methods will not receive chemotherapy, while those considered high risk for relapse by both methods will be given the opportunity to receive adjuvant chemotherapy. Patients of primary interest, those with discordant results, will be randomized to treatment based on either Adjuvant! Online or MammaPrint^™^ to determine which test is more effective in defining treatment in node-negative patients. The Trial Assigning Individualized Options for Treatment (TAILORx) is examining whether hormone receptor-positive patients with an intermediate Onco*type* DX^™^ risk recurrence score benefit from chemotherapy. The trial is recruiting 10,000 hormone-receptor-positive patients with HER2-negative and lymph-node-negative disease. Treatment will be based on the risk recurrence score as follows: ≤10 — hormone therapy alone; ≥26 — hormone and chemotherapy; intermediate scores — randomization to either hormone therapy alone or to hormone therapy and chemotherapy. The goal is to integrate Onco*type* DX^™^ into the clinical decision-making process and refine the utility of the assay in clinical practice [111].

Molecular signatures have improved the ability to predict outcome and identify breast cancer patients who would most likely benefit from systemic therapy, thus providing an additional layer of personalized medicine. However, no current molecular signature is 100% accurate, and 5-10% of patients now classified as low-risk are likely to relapse. Furthermore, current classification systems were developed to predict only short-term (<5 years) outcomes; thus there is a need to develop signatures that identify patients with protracted disease progression who may benefit from prolonged therapy [112]. Although outcome prediction tends to be similar between gene-expression signatures, overlap among genes comprising the signatures is relatively low, suggesting that these profiles assess common biological pathways, but have not identified the actual genes driving tumor behavior and outcome [113]. Finally, some multigene predictor assays are being adopted and marketed before they have been properly validated and proven to be clinically informative, thus the degree to which expression-based tests will alter the course of patient treatment remains unclear [114, 115].

## PHARMACOGENOMICS OF BREAST CANCER

Pharmacogenomics in breast cancer evaluates the effect of inherited genomic variation on patient response or resistance to treatment. Genetic variability is commonly measured at the DNA level in the form of chromosomal alterations or DNA sequence variants (Table **4**). Conversely, somatic genomic changes (DNA variants and gene expression profiles) in breast tumors can influence rates of apoptosis, cell proliferation, and DNA damage repair, which may have direct effects on response to treatment and survival. To be most effective, personalized medicine must incorporate information from innate genetic variation as well as somatic mutations in diseased tissue [116].

### Endocrine Therapy 

Estrogens play an important role in the etiology of breast cancer by stimulating growth and proliferation of ductal epithelial cells in the breast, thus the status of the estrogen receptor in breast carcinomas provided one of the earliest avenues for personalized medicine. Fortunately, hormone-receptor-positive tumors usually are responsive to agents such as Tamoxifen that block the function of estrogen. Tamoxifen is a potent antagonist of the ER with inhibitory effects on tumor growth that has become the gold standard for endocrine treatment of estrogen-receptor-positive breast cancer in premenopausal and postmenopausal women [117]. Tamoxifen is associated with side effects such as blood clots, stroke, and increased risk of endometrial and uterine cancer, but five-year use of tamoxifen has been shown to reduce risk of cancer recurrence by ~50% [118]. For most patients, the benefit of using tamoxifen for hormone-receptor-positive disease outweighs the risk of serious side effects; however, a small subgroup of hormone-receptor-positive patients who carry specific variants in the cytochrome P450 2D6 (CYP2D6) gene do not benefit from tamoxifen. The CYP2D6 gene is a key enzyme in the metabolism of tamoxifen to its active metabolite endoxifen. Several DNA variants in CYP2D6 result in poor metabolism of tamoxifen and lower levels of endoxifen [119]. Patients who carry reduced-function or nonfunctional CYP2D6 alleles have been found to derive inferior therapeutic benefit from tamoxifen and thus are at increased risk of breast cancer recurrence [120] or have significantly shorter disease-free survival than non-carriers [121]. Studies are underway to determine the utility of CYP2D6 genotyping for making clinical decisions about tamoxifen and the potential to optimize breast cancer therapy [122, 123].

Alternate forms of directed anti-estrogen therapies do exist for patients with hormone-receptor-positive breast cancer including aromatase inhibitors that block the production of estrogen, and compounds such as fulvestrant (Faslodex^®^) that down-regulate and degrade the ER protein. Aromatase inhibitors such as anastrozole (Arimidex^®^), letrozole (Femara^®^), and exemestane (Aromasin^®^) target cytochrome P450 19 (CYP19A1 or aromatase), an enzyme involved in estrogen synthesis in peripheral organs. Premenopausal women with functional ovaries do not receive aromatase inhibitor therapy because first and second generation aromatase inhibitors did not effectively suppress estrogen levels and because decreased estrogen levels in peripheral tissues could be counteracted by increased estrogen synthesis in the ovaries [124]. In postmenopausal women, aromatase inhibitors are well-tolerated and improve both disease-free and recurrence-free survival [125-127]. Similar to CYP2D6, the Cys^264^ and Thr^364^ variants in aromatase are associated with decreased activity and lower levels of immunoreactive protein, which may contribute to variation among patients in response to aromatase inhibitor therapy [128]. Although directed endocrine therapies provide treatments specific for patients with hormone-receptor-positive breast cancer, factors such as menopausal status and innate genetic variability may alter the effectiveness of treatment.

### Treatment for HER2 Positive Breast Cancer

Therapies directed at the HER2 protein provide a second avenue of targeted treatment for some patients with breast cancer. Trastuzumab (Herceptin^®^, Genentech, South San Francisco, CA; www.gene.com/) is a humanized monoclonal antibody that binds to the extracellular domain of the HER2 protein, blocking tumor cell growth. Trastuzumab is the current standard of care in adjuvant therapy for HER2-positive breast cancer, effective as a single agent or in combination with chemotherapeutics for the 20-25% of patients with HER2-positive cancer [129]. However, many patients with HER2-positive disease do not derive tangible benefit from trastuzumab. Given that the cost per patient for trastuzumab ranges from $20,000-$80,000 per year with the potential for significant adverse side effects [130], a more precise classification of HER2-positive patients who will derive benefit from trastuzumab and improved understanding of how amplification and/or over-expression of HER2 contribute to aggressive tumor biology are critical to improving patient treatment.

The major oncogenic unit in HER2-positive breast cancer appears to be a heterodimer between the HER2 and epidermal growth factor receptor-3 (HER3) proteins, where HER3 functions as a necessary dimerization partner for HER2 to achieve full oncogenic signaling potential [131]. Recent studies have shown that HER2/HER3 heterodimers promote cellular proliferation in both *in vitro* and *in vivo* models, suggesting that HER3 may be an important therapeutic target in HER2-positive patients [132]. Pertuzumab has been shown to bind to the dimerization arm of HER2, blocking HER2/HER3 heterodimerization and attenuating growth of solid tumors in model systems [133]. Thus, combining pertuzumab with trastuzumab may augment therapeutic benefit by blocking HER2/HER3 signaling. Monogram Biosciences (South San Francisco, CA; www.monogrambio.com/) has developed the commercially-available HERmark^™^ test to measure total HER2 levels and HER2 homodimers in FFPE tissue and is developing a *VeraTag*^™^ assay to quantify levels of HER2/HER3 heterodimers. These assays may allow patients with HER2-positive breast cancer to receive the most efficacious combination of new drugs targeting HER2.

### Chemotherapeutics

Chemotherapy involves use of chemical agents as part of a systemic treatment targeting proliferative cancer cells. Adjuvant chemotherapy is used to reduce risk of recurrence after primary therapy in women with localized breast cancer and to provide palliative care in patients with advanced (metastatic) disease. In contrast, neoadjuvant chemotherapy is normally used to shrink moderate- to large-sized breast carcinomas prior to surgical resection, which permits use of less aggressive surgical options, including breast conservation, and may be useful in guiding longer-term treatment based on tumor response to specific drug combinations [134]. Obviously, the ability to predict which patients will benefit from adjuvant therapy and identify who will respond favorably to neoadjuvant regimens would provide an additional level of personalized care.

#### Gene Expression and Chemotherapeutic Agents

Gene expression profiling has been used to study the biological responses of human breast carcinomas to optimize chemotherapeutic treatments. Cell lines derived from luminal and basal epithelium have been observed to respond differently to agents commonly used in chemotherapy, such as doxorubicin (DOX) and 5-fluorouracil (5FU). In culture, luminal cell lines show low levels of expression for genes regulating cellular proliferation and the cell cycle, while basal cell lines tend to repress genes involved in cellular differentiation when exposed to DOX and 5FU [135]. Similarly, different molecular subtypes of breast cancer defined by gene expression profiling respond differently to preoperative chemotherapy, with basal-like and HER2-positive subtypes being more sensitive to paclitaxel and doxorubicin than luminal and normal-like cancers [136]. Expression signatures also have been used to predict clinical response of breast cancer patients receiving either cyclophosphamide-adriamycin or epirubicin-5FU as part of their adjuvant chemotherapy regimen [137] and to distinguish primary breast tumors that are responsive or resistant to docetaxel chemotherapy [138]. These observations further highlight the vast amount of molecular variability among breast carcinomas and emphasize the need for additional molecular signatures to more effectively guide treatment.

#### DNA Variation and Chemotherapeutic Agents

Clinical responses in breast cancer patients to commonly used chemotherapeutic agents vary considerably, from optimum therapeutic response to partial (beneficial) response to severe adverse events. Variation at the DNA level in an increasing number of genes is now known to affect the pharmacokinetics and pharmacodynamics of many chemotherapeutic drugs [139, 140], thus influencing toxicity and patient response. To improve the safety and efficacy of current treatments, therapies could be tailored to individual patients based on their genetic makeup [141]. For example, the carbonyl reductase 3 (CBR3) gene contributes to the reduction of DOX to doxorubicinol, a less potent metabolite, and the extent of metabolism is believed to be a source of variability in doxorubicin chemotherapy. The 11G>A variant (rs8133052) in CBR3 has been shown to influence tumor tissue expression of CBR3 and is associated with inter-individual variability in clinical outcomes. Women with the 11GG genotype experience greater leukocyte toxicity and are less likely to show a reduction in tumor size than women carrying 11AA [142].

A number of chemotherapeutics generate reactive oxygen species that function by damaging DNA and triggering the apoptotic cascade. Women carrying variants in genes associated with oxidative stress, such as manganese superoxide dismutase (MnSOD), catalase (CAT), and myeloperoxidase (MPO) that result in higher levels of reactive oxygen species, tend to have better overall survival than women with genotypes associated with lower levels of reactive oxygen species when treated with chemotherapy [143]. Due to the large number of drug-metabolizing enzymes and drug transporters containing polymorphisms that affect chemotherapy-related toxicity and treatment outcomes in breast cancer patients, improved pharmacogenetic information is needed to identify individuals at risk for toxicity and poor response.

### Genomics in Clinical Practice

Recent developments in the clinical arena are indicative of the emerging importance of personal genomics in the prevention, surveillance, and treatment of breast cancer. Professional organizations such as the American Society of Clinical Oncology (ASCO) have issued recommendations on the use of molecular markers for guiding therapy and determining prognosis in breast cancer patients [144]:

- CA 15-3 and CA 27.29 (assays to detect circulating MUC-1 antigen in peripheral blood) — contributes to decisions regarding therapy for metastatic breast cancer in conjunction with diagnostic imaging, history, and physical examination
- Carcinoembryonic antigen (CEA) — contributes to decisions regarding therapy for metastatic breast cancer in conjunction with diagnostic imaging, history, and physical examination
- ER/PR — should be measured on every primary invasive breast cancer to identify patients most likely to benefit from endocrine therapy
- HER2 — should be measured on every primary invasive breast cancer at diagnosis or recurrence to guide trastuzumab therapy
- Urokinse plasminogen activator (uPA) and plasminogen activator inhibitor 1 (PAI-1) — measured by ELISA on fresh or frozen tissue for determining prognosis in newly-diagnosed, node-negative breast cancer patients
- Onco*type* DX^®^ — in newly-diagnosed patients with node-negative, ER-positive breast cancer, can be used to predict risk of recurrence in women treated with tamoxifen

Large cancer centers such as Massachusetts General Hospital (MGH) and Memorial Sloan-Kettering Cancer Center (MSKCC) are now embracing the importance of genomics in clinical practice, recently implementing policies to routinely assay a number of breast cancer-related genes — vakt murine thymoma viral oncogene homolog 1 (AKT1) and HER2 at MSKCC, phosphatase and tensin homolog (PTEN) and TP53 at MGH, and phosphatidylinositol 3-kinase, catalytic, alpha (PIK3CA) at both institutions [145]. As genomic medicine becomes an integrated part of health care delivery, use of personalized genomics in the clinical treatment of breast cancer will increase.

## CONCLUSIONS

The era of personalized molecular medicine for breast cancer is on the horizon. Identification of strongly penetrant genes such as BRCA1 and BRCA2 has improved risk assessment for women with hereditary forms of breast cancer, but sporadic breast cancer presents additional challenges due to the influence on disease risk of lifestyle and environmental factors as well as common low-risk DNA variants. Substantial progress has been made in applying genomic discoveries to breast cancer treatment, as gene expression profiles are now being used to partition heterogeneous breast carcinomas into specific groups associated with different prognosis, pathological features, and developmental behavior. Customized treatments based on genetic susceptibility of the patient and molecular characteristics of the tumor allow more effective treatments that minimize adverse drug reactions. Yet despite these advances, personalized genomics in breast cancer is still in its infancy and genomic technologies have just begun to realize their full potential in clinical practice. Currently, the majority of genes contributing to sporadic breast cancer have not been identified. Whole-genome association studies have identified some DNA variants contributing to breast cancer risk that provide new insights into the pathophysiology of disease and may ultimately prove useful for developing targeted interventions [146], but many variants will never have clinical utility. Although molecular information may estimate risk and guide treatment in certain populations, expression profiles may not be applicable to other high-risk groups. To maximize the effectiveness of genomic data, other factors that influence breast cancer risk, such as the impact of environmental exposures, lifestyle factors, and personal behaviors must become integral components of risk prediction models.

Recently proposed health-care reform legislation does not advocate personalized genomic medicine directly, but has the potential to profoundly affect genomics in medicine by adopting a shared decision making model that would incorporate patient preferences and values into their medical treatment plan. As educated patients become more knowledgeable about personalized genomics, demand for molecular-based tests may increase dramatically. Expanded use of genetic information for tailoring treatments in breast cancer patients will present several challenges, such as managing the impact of genetic testing on healthcare delivery and cost [147]. In order to maximize the potential of personal genomics in medicine, physicians must be fully prepared to deal with issues related to genomic tests, including the ability to critically evaluate and interpret genomic results [148] and issues such as cost-effectiveness, predictive limitations, and impact on quality of life must be considered.

## Figures and Tables

**Fig. (1):**
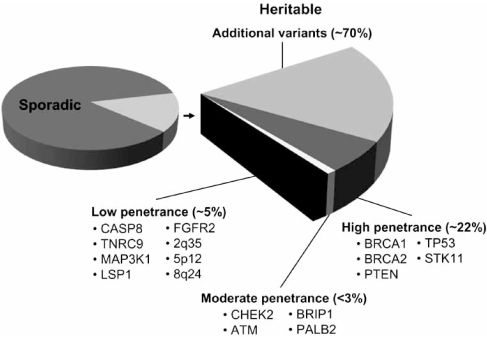
Complexity and largely unknown molecular etiology of breast cancer. The majority of breast cancer cases (~70%) are considered sporadic in nature because individuals with disease do not have an extensive family history of breast cancer and molecular alterations contributing to the disease have not been identified. Familial breast cancer (~30% of patients), often seen in families with a high incidence of breast cancer, has been associated with a number of high-, moderate-, and low-penetrance susceptibility genes.

**Table 1:** Low-Penetrance Variants that may Influence Sporadic Breast Cancer Identified through Genome-Wide Association Studies

SNP	Chromosome	Candidate Genes	MAF^a^	OR^b^	Reference
rs889312	5q11	MAP3K1	0.28	1.13	[60]
rs2180341	6q22	ECHDC1, RNF146	0.27	1.41	[63]
rs2981582	10q26	FGFR2	0.38	1.26	[60, 61]
rs3803662	16q12	TNRC9, LOC643714	0.25	1.20	[60, 62]
rs3817198	11p15	LSP1	0.30	1.07	[60]
rs10941679	5p12	MRPS30	0.25	1.19	[64]
rs13281615	8q24		0.40	1.08	[60]
rs13387042	2q35		0.50	1.21	[62]

a MAF = minor allele frequency.

b OR = odds ratio per allele.

**Table 2:** Leading Direct-to-Consumer Genetic Testing Companies

Company	Headquarters	Website	Cost (USD)	Genetic Counseling	Breast Cancer Susceptibility Variants
23andMe	Mountain View, CA	www.23andme.com	$399	No	2 SNPS
deCODEme	Reykjavik, Iceland	www.decodeme.com	$985^a^	Yes	11 variants^b^
Knome	Cambridge, MA	www.knome.com	Custom^c^	Yes	DNA sequence
Navigenics	Foster City, CA	www.navigenics.com	$999^d^	Yes	unknown

a Complete scan.

b For women of European descent.

c KnomeSELECT^™^ is $24,500 for complete sequence of 20,000 genes; KnomeCOMPLETE^™^ is $99,500 for complete genome sequence.

d Option for ongoing subscription ($199 per year) for updates.

**Table 3:** Selected Molecular Diagnostic Tests for Breast Cancer

Test	Company	Assay Type^a^	Number of Genes/Proteins	Classification	Reference
Breast Bioclassifier^™^	University Genomics	qRT-PCR	55	Tumor subtype Therapeutic guidance	[9]
MammaPrint^™^	Agendia	Microarray	70	Prognostic Therapeutic guidance	[105, 106]
MammoStrat^®^	Applied Genomics	IHC	5	Prognostic	[97]
Map*Quant* DX^™^	Ipsogen	Microarray	97	Tumor grade	[103, 104]
Onco*type* DX^™^	Genomic Health	qRT-PCR	21	Prognostic Therapeutic guidance	[108, 109]
Rotterdam signature	Veridex	Microarray	76	Prognostic	[107]

a qRT-PCR = quantitative real-time PCR; IHC = immunohistochemistry.

**Table 4:** Selected Genetic Polymorphisms Affecting Response to Therapy in Breast Cancer Patients

Treatment	Gene	Variant	Functional Change	Response to Treatment	Reference
Chemotherapy					
Doxorubicin	CBR3	11G>A	Decreased enzyme activity	Hematological toxicity	[142]
Anthracyclines	MnSOD	Ala^16^	Higher levels of reactive oxygen species	Decreased mortality	[143]
	MPO	—463GG	Higher levels of reactive oxygen species	Decreased mortality	[143]
	GSTP1	313A>G	Altered drug transport	Hematological toxicity	[150]
	MTHFR	1298A>C	Altered drug metabolism	Non-hematological toxicity	[150]
Endocrine therapy					
Tamoxifen	CYP2D6	*3, *4, *5, *10, *41	Reduced function/nonfunctional enzyme	Poor clinical outcome	[120, 121]
Aromatase inhibitors	CYP19A1	Cys^264^, Thr^364^	Decreased enzyme activity	Reduced benefit	[128]
Radiotherapy					
	TP53	Arg72Pro, PIN3	Decreased apoptosis	Risk of telangiectasia	[149]
Targeted therapy					
Trastuzumab	HER2	Heterodimer	Prevents disruption by trastuzumab	Poor response to treatment	[132]
